# Community Understanding and Attitudes Toward Prostate-Specific Antigen (PSA) Testing for Prostate Cancer Screening: A Cross-Sectional Survey Study From Saudi Arabia

**DOI:** 10.7759/cureus.97658

**Published:** 2025-11-24

**Authors:** Ali F Alkhars, Abdullatif K Almaghlouth, Nawaf F Almusalem, Mohammed S Alobud, Abdulelah B Alshafei, Mohammed S Al Janobi, Hosain A Al Watyan, Hisham M Alramadhan, Hassan A Alramadhan

**Affiliations:** 1 College of Medicine, King Faisal University, Al Ahsa, SAU; 2 Urology, King Faisal University, Al Ahsa, SAU; 3 College of Medicine, AlMaarefa University, Al Ahsa, SAU

**Keywords:** attitudes, cross-sectional studies, early detection of cancer, health education, health surveys, practice, prostate cancer, prostate-specific antigen, risk factors, saudi arabia

## Abstract

Background: Prostate cancer (PC) significantly impacts men’s health globally, including in Saudi Arabia. This study evaluates public awareness of prostate-specific antigen (PSA) testing, identifies barriers to screening participation, and explores factors influencing knowledge levels in Al Ahsa, Saudi Arabia.

Methods: A cross-sectional, self-administered electronic survey was conducted among 388 participants. Convenience sampling was utilized within a population of approximately 1.3 million. The Arabic questionnaire, adapted from a prior study, assessed demographic data, knowledge, and attitudes toward PC and PSA screening. Statistical analysis included descriptive statistics and chi-square or Fisher’s exact tests, with significance set at p<0.05.

Results: Most respondents were aged 18-28 (75.3%, 292 participants), unmarried (71.6%, 278 participants), and possessed bachelor’s degrees (48.7%, 189 participants). Although 62.4% (242 participants) recognized PC as the most common malignancy in men, only 29.1% (113 participants) were aware of PSA testing. A mere 5.9% (23 participants) had previously undergone PSA testing, despite 57.4% (223 participants) acknowledging age over 50 as a risk factor. Notably, 93.3% (362 participants) had never sought medical assistance for prostate issues, with 79.9% (310 participants) expressing the need for additional information. Employment status and educational attainment significantly influenced knowledge levels (p=0.004 and p=0.003).

Conclusion: There is a substantial lack of knowledge and awareness about PC and PSA screening among Saudi participants. Barriers include low perceived risk and fear of diagnosis, compounded by reliance on informal sources such as social media. Addressing these gaps through targeted education and proactive outreach initiatives is crucial for enhancing awareness and encouraging early screening behaviors.

## Introduction

Prostate cancer (PC) is a significant health concern for all men, particularly those over the age of 40, as it poses considerable health risks [1]. Over the past decade, the implementation of screening strategies for PC has markedly influenced both morbidity and mortality rates [1]. Screening aims primarily to identify PC in asymptomatic individuals, with digital rectal examination (DRE) and blood prostate-specific antigen (PSA) testing serving as foundational diagnostic tools [2].

Globally, PC ranks as the second leading cause of cancer-related mortality among men. According to the American Cancer Society’s 2019 statistics, approximately 174,650 new cases of PC were diagnosed in the United States during that year, with an estimated lifetime mortality risk of one in every 41 men [3]. Despite its widespread prevalence, the underlying etiology of PC remains inadequately understood beyond well-established risk factors, including advancing age, a familial history of the disease, Lynch syndrome, and mutations in genes such as BRCA1 and BRCA2, which are critical for DNA repair [4]. Furthermore, lifestyle and biological factors, including smoking, obesity, and elevated testosterone levels, have been implicated in the disease’s progression [4].

In Saudi Arabia, PC is the sixth most frequently diagnosed malignancy among men [5]. However, awareness regarding PC and its screening methods remains limited [6]. A 2015 study conducted in Riyadh identified various contributing factors to this gap, including cultural and societal barriers, insufficient public health campaigns, and inadequate physician counseling [1]. In a separate study in Jazan, 52.9% of participants were likely to disregard medical advice and forgo PSA testing, while PSA testing was preferred by 6.8% of participants compared to 3.2% for DRE as a screening method [7].

Although DRE and PSA testing remain integral to PC screening, this study prioritizes PSA testing due to its broader public acceptance and less invasive nature in the local population. This study aims to measure public awareness of PSA testing in Al Ahsa, Saudi Arabia, identify barriers that may hinder participation in PC screening, and examine the factors that influence knowledge levels within the community.

## Materials and methods

Study design

A cross-sectional, electronic, self-administered survey study was deemed suitable as it required systematic data collection from a large, geographically dispersed group of participants.

Population and sampling

The sample size for the study was calculated using the online tool calculator.net, resulting in 385 participants. The target population was estimated at 1.3 million. This sample size was based on a 50% response distribution, a 95% CI, and a 5% margin of error. We collected 388 responses. A non-probability convenience sampling technique was employed to select participants who met the inclusion criteria and agreed to participate in the study.

The inclusion criteria included individuals aged 18 years and above who were residing in or originally from Al Ahsa city in Saudi Arabia. Those below 18 years, from other towns, or anyone who declined to participate were excluded from the study.

Data collection

After conducting a thorough literature review and consulting experts in the field, the researchers used a validated, pre-structured questionnaire adapted from a study by Morlando et al. [8] to collect data. Twenty-five males participated in a pilot study of the questionnaire, which was assessed for reliability (Cronbach's α=0.72). During this pilot phase, participants provided detailed feedback on each item's comprehensiveness, relevance, and clarity. Based on their suggestions, several questions were revised to reduce ambiguity and enhance readability.

Google Forms was used to create an online survey that included an introductory page outlining the study objectives, inclusion criteria, and the voluntary nature of participation. Participants provided informed consent electronically before accessing the questionnaire. The survey collected demographic information, personal and family history of PC, knowledge of PC and PSA testing, perceived risk, opinions on PSA testing, willingness to be tested, sources of information, and informational needs regarding PC and PSA testing.

After obtaining approval from King Faisal University's institutional research board, the survey was distributed electronically via email and social media to all participants who met the inclusion criteria. No schools or universities were specifically targeted. Multiple platforms were used to maximize reach, including WhatsApp community groups and local social media networks.

Before analysis, responses were screened to ensure completeness, and Google Forms’ duplicate-response restriction (one response per account) was enabled to reduce the likelihood of repeated submissions.

The study commenced in October 2024 and concluded in April 2025.

Data analysis

Data were analyzed using IBM SPSS Statistics for Windows, Version 27.0 (IBM Corp., Armonk, NY, USA). Descriptive statistics summarized the sociodemographic characteristics and health-related data of the participants. Categorical variables were presented as frequencies and percentages, while continuous variables were expressed as means and SD.

Participants' knowledge of PC and PSA testing was assessed by assigning one point for each correct answer in the knowledge section of the questionnaire. The total knowledge score was calculated with a possible range from 0 to 10 points, based on the number of knowledge items provided. A cutoff point of 60% was used to categorize knowledge levels: participants scoring less than 60% (<6 points) were considered to have poor knowledge, while those scoring 60% or higher (≥6 points) were regarded as having good knowledge.

The Chi-square test was utilized for categorical variables to examine the associations between participants' knowledge levels and various factors such as age, marital status, educational level, employment status, health condition, family history, and prostate health practices. When the expected frequency in any cell was less than 5, the Exact probability test (Fisher's Exact test) was applied instead. A p-value of less than 0.05 was considered statistically significant for all analyses.

## Results

The sociodemographic and health-related details of the Saudi Arabian participants in the PSA testing awareness study are shown in Table 1. There were smaller percentages in the older age groups: 33 (8.5%) were between the ages of 29 and 39, 32 (8.2%) were between 40 and 50, 24 (6.2%) were between 51 and 60, and only seven (1.8%) were over 60. The majority of participants (292; 75.3%) were between 18 and 28 years of age. Regarding marital status, 278 (71.6%) participants were single, while 110 (28.4%) were married. Regarding educational level, 189 (48.7%) participants held a bachelor’s degree, 124 (32.0%) had completed secondary education, 51 (13.1%) earned a diploma, 14 (3.6%) had below secondary education, and 10 (2.6%) held postgraduate qualifications. The participants’ employment status showed that 12 (3.1%) were retired, 45 (11.6%) were unemployed, 127 (32.7%) were employed, and 204 (52.6%) were students. Two hundred seventy participants (69.6%) rated their current health status as good, 104 (26.7%) as intermediate, and 14 (3.6%) as poor. Furthermore, 316 (81.4%) of the participants did not report a family history of prostatic disease, whereas 72 (18.6%) did.

**Table 1:** Sociodemographic characteristics and health-related data of study participants in Saudi Arabia (N=388)

Data	No	%
Age in years		
18-28	292	75.3%
29-39	33	8.5%
40-50	32	8.2%
51-60	24	6.2%
>60	7	1.8%
Marital status		
Single	278	71.6%
Married	110	28.4%
Educational level		
Below secondary	14	3.6%
Secondary	124	32.0%
Diploma	51	13.1%
Bachelor's degree	189	48.7%
Post-graduate	10	2.6%
Employment		
Unemployed	45	11.6%
Student	204	52.6%
Employee	127	32.7%
Retired	12	3.1%
How would you rate your current health condition		
Poor	14	3.6%
Intermediate	104	26.8%
Good	270	69.6%
Mean±SD (health condition)	8.2±1.7	
Family history of prostatic disease		
Yes	72	18.6%
No	316	81.4%

Participants’ awareness and knowledge of PSA testing and PC are described in Table 2. Most participants (242; 62.4%) were aware that the most common malignant tumor in men is PC. One hundred sixty-four (86.8%) of the nearly half (189; 48.7%) who reported receiving information about PC thought the information was helpful. However, a significant proportion (310; 79.9%) expressed a need for more information on PC. Regarding risk factors, age over 50 years (218; 57.4%), smoking (191; 50.3%), and family history (183; 48.2%) were the most commonly identified. Most participants (262; 67.5%) correctly recognized that men over 50 years are at the highest risk of developing PC. Physical activity (213; 54.9%) and a low-fat diet (167; 43.0%) were the most frequently mentioned preventive measures. Regarding PSA knowledge, only 113 (29.1%) participants had heard of the PSA, and an even smaller fraction (83; 21.4%) had received information about PSA testing. Among those who received information, 77 (92.8%) found it helpful. Similar to knowledge about PC, a large majority (307; 79.1%) voiced a need for more information on PSA testing.

**Table 2:** Participant knowledge and awareness of PC and PSA testing (N=388) PSA, prostate-specific antigen; PC, prostate cancer

Domain	Knowledge items	No	%
PC knowledge	PC is the most common malignant tumor in men. Have you ever heard of that?		
	Yes	242	62.4%
	No	146	37.6%
	Have you ever received information about PC?		
	Yes	189	48.7%
	No	199	51.3%
	Was the information you received about PC helpful?		
	Yes	164	86.8%
	No	25	13.2%
	Do you need more information about PC?		
	Yes	310	79.9%
	No	78	20.1%
	What factor may make it more likely to develop PC?		
	Age above 50 years	218	57.4%
	Smoking	191	50.3%
	Family history	183	48.2%
	High-fat diet	121	31.8%
	Alcohol	107	28.2%
	Obesity	104	27.4%
	I do not know	94	24.7%
	Number of sexual partners	64	16.8%
	At what age are men most at risk of developing PC?		
	Above 50 years	262	67.5%
	Below 50 years	18	4.6%
	I do not know	108	27.8%
	What can prevent the onset of PC?		
	Physical activity	213	54.9%
	Low-fat diet	167	43.0%
	Fruits and vegetables (no less than five servings per day)	153	39.4%
	Vitamin D/E	131	33.8%
	Meat	34	8.8%
	Butter	21	5.4%
	I do not know	152	39.2%
PSA knowledge	Previously heard about PSA		
	Yes	113	29.1%
	No	275	70.9%
	Have you ever received information about PSA testing?		
	Yes	83	21.4%
	No	305	78.6%
	Was the information you received about PSA testing helpful?		
	Yes	77	92.8%
	No	6	7.2%
	Do you need more information about PSA testing?		
	Yes	307	79.1%
	No	81	20.9%

**Figure 1:**
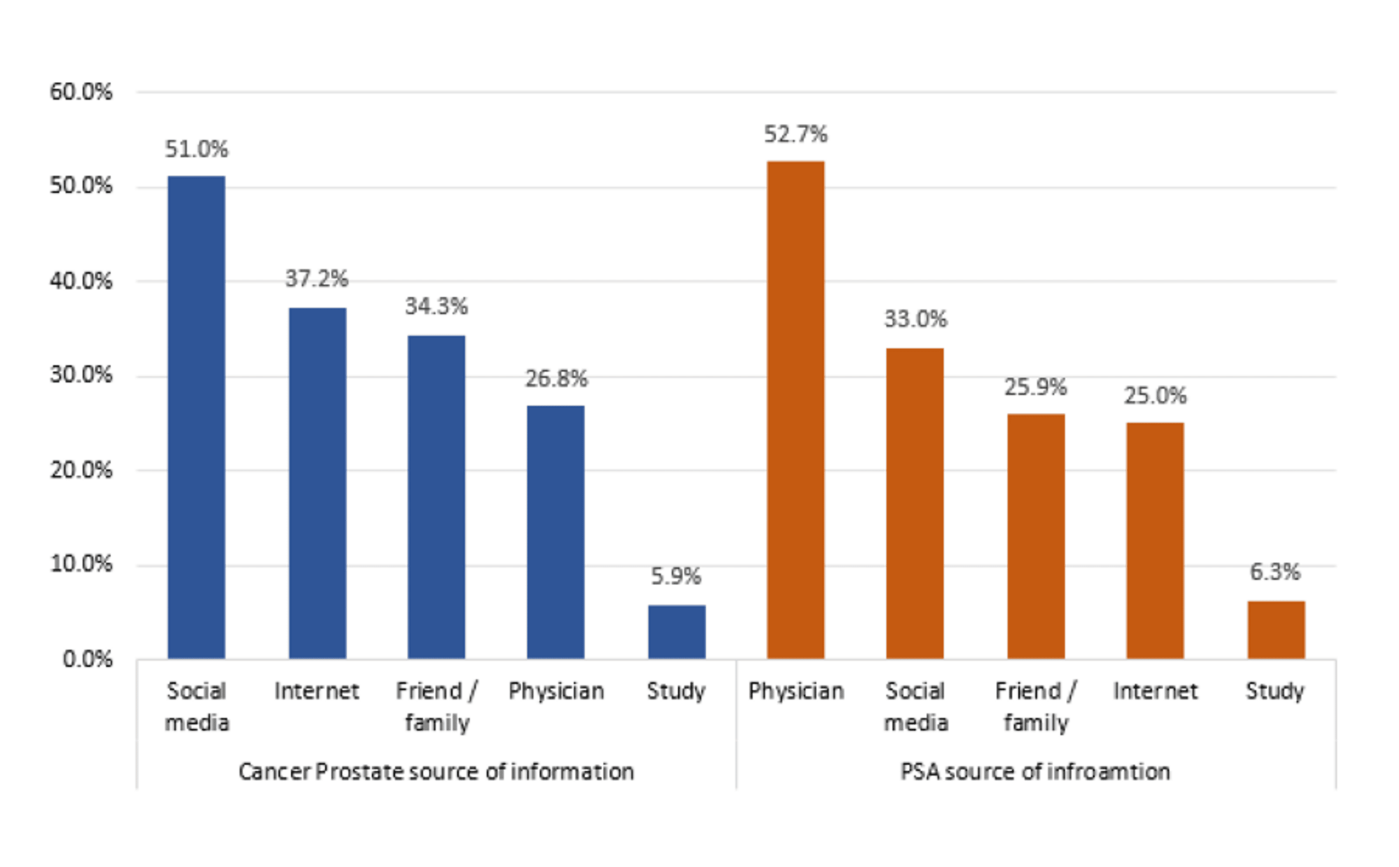
Source of information about PC and PSA among study participants, Saudi Arabia (N=388) PSA, prostate-specific antigen; PC, prostate cancer

In terms of overall knowledge level, the majority of participants (292; 75.3%) displayed a poor level of understanding, while 96 (24.7%) demonstrated a good level of expertise. Regarding sources of information on PC (Figure 1), social media was the most commonly cited source (122; 51.0%), followed by the internet (89; 37.2%) and friends or family (82; 34.3%). Physicians served as a source of information for 64 (26.8%) participants, whereas formal studies were the least common (14; 5.9%). Among those who received information specifically about PSA testing, physicians were regarded as the most trusted source (59; 52.7%), followed by social media (37; 33.0%), friends or family (29; 25.9%), and the internet (28; 25.0%). Formal studies continued to be the least common source (seven; 6.3%).

Table 3 reveals participants’ attitudes toward PC risk and the importance of PSA testing. Most participants (270; 69.6%) agreed that the possibility of developing PC increases with age, while 100 (25.8%) were unsure, and 18 (4.6%) disagreed. Regarding PSA testing, 264 (68.0%) participants agreed that asymptomatic men over 50 years should undergo a PSA test, while 106 (27.3%) were unsure, and 18 (4.6%) disagreed. Furthermore, 285 (73.5%) participants recognized that one benefit of PSA testing is detecting cancer before symptoms appear. However, 93 (24.0%) remained unsure. When asked about personal concerns, 121 (31.2%) expressed worry about getting PC, while 165 (42.5%) disagreed, and 102 (26.3%) were unsure.

**Table 3:** Participant attitudes toward PC risk and PSA testing (N=388) PSA, prostate-specific antigen; PC, prostate cancer

Attitude items	Agree		Not sure		Disagree	
	No	%	No	%	No	%
The possibility of developing PC increases with age	270	69.6%	100	25.8%	18	4.6%
Asymptomatic men over the age of 50 should undergo a PSA test	264	68.0%	106	27.3%	18	4.6%
One of the benefits of PSA testing is to detect cancer before symptoms appear	285	73.5%	93	24.0%	10	2.6%
I am worried about getting PC	121	31.2%	102	26.3%	165	42.5%

Table 4 outlines the practices and barriers preventing participants from undergoing PSA testing. The vast majority (362; 93.3%) reported never having visited a doctor or urologist for prostate-related issues, and only 26 (6.7%) had done so. Among those who visited a doctor, only nine (34.6%) were informed about the PSA test, while 17 (65.4%) were not. Regarding PSA testing, only 23 (5.9%) participants reported having undergone the test, while 365 (94.1%) had not. Among those who had the test, the primary reasons included being advised to do so (13; 54.2%), feeling at risk (12; 50.0%), and participating in prevention programs (10; 41.7%). Only five (20.8%) underwent the test specifically to detect PC before symptoms appeared. When asked if they would consider a future PSA test, 223 (57.5%) responded positively, with early detection (167; 72.9%) being the primary reason. However, 165 (42.5%) expressed reluctance, citing not feeling at risk (120; 72.7%), fear of discovering PC (15; 9.1%), or believing the test is not useful (11; 6.7%). A small proportion (seven; 4.2%) reported being deterred by their doctor.

**Table 4:** Practice and barriers to PSA testing among participants (N=388) PSA, prostate-specific antigen; PC, prostate cancer

Practice	No	%
Have you ever gone to a doctor and/or urologist for prostate problems?		
Yes	26	6.7%
No	362	93.3%
Has your doctor and/or urologist told you about the PSA test?		
Yes	9	34.6%
No	17	65.4%
Have you ever had a PSA test?		
Yes	23	5.9%
No	365	94.1%
If yes, why did you undergo the test?		
I was advised after undergoing it	13	54.2%
Participated in prevention programs	10	41.7%
I felt danger	12	50.0%
To detect PC before symptoms appear	5	20.8%
If it were you, would you have a PSA test?		
Yes	223	57.5%
No	165	42.5%
If yes, why?		
To detect PC before symptoms appear	167	72.9%
After talking to my physician	87	38.0%
Feel danger	48	21.0%
To be safe	5	2.2%
If not, Why?		
I don't feel like I'm in danger	120	72.7%
Others	58	35.2%
Afraid of discovering PC	15	9.1%
Because it is not useful	11	6.7%
The doctor deterred me	7	4.2%

Table 5 examines factors associated with participants’ knowledge levels about PC and PSA testing. Age showed no significant association with knowledge levels (p=0.320), with poor knowledge consistently high across all age groups, ranging from 71.9% (40-50 years) to 100% (>60 years). Marital status also showed no significant association (p=0.271), with 73.7% of single participants and 79.1% of married participants having poor knowledge. However, educational level was significantly associated with knowledge (p=0.004), as participants with a bachelor’s degree (70.4% poor knowledge) and postgraduate education (60.0% poor knowledge) demonstrated better knowledge compared to those with a diploma (96.1% poor knowledge). Employment status also showed a significant association (p=0.003), with students having the lowest proportion of poor knowledge (68.1%) and retirees having the highest (100.0%). Self-rated health and family history of prostatic disease were not significantly associated with knowledge levels (p=0.278 and p=0.335). Prior visits to a doctor or urologist, receiving information about PSA testing, and having undergone a PSA test were not significantly associated with knowledge (p=0.790, p=0.143, and p=0.878). Willingness to undergo future PSA testing showed no significant association with knowledge levels (p=0.363).

**Table 5:** Factors associated with participants’ knowledge about PC and PSA ^Exact probability test *P<0.05 (significant) P, Pearson X2 test; PSA, prostate-specific antigen; PC, prostate cancer

Factor	Overall knowledge level				p-value
	Poor		Good		
	No	%	No	%	
Age in years					0.320
18-28	216	74.0%	76	26.0%	
29-39	25	75.8%	8	24.2%	
40-50	23	71.9%	9	28.1%	
51-60	21	87.5%	3	12.5%	
Above 61	7	100.0%	0	0.0%	
Marital status					0.271
Single	205	73.7%	73	26.3%	
Married	87	79.1%	23	20.9%	
Educational level					0.004*^
Below secondary	11	78.6%	3	21.4%	
Secondary	93	75.0%	31	25.0%	
Diploma	49	96.1%	2	3.9%	
Bachelor's degree	133	70.4%	56	29.6%	
Post-graduate	6	60.0%	4	40.0%	
Employment					0.003*
Unemployed	38	84.4%	7	15.6%	
Student	139	68.1%	65	31.9%	
Employee	103	81.1%	24	18.9%	
Retired	12	100.0%	0	0.0%	
How would you rate your current health condition?					0.278
Poor	13	92.9%	1	7.1%	
Intermediate	79	76.0%	25	24.0%	
Good	200	74.1%	70	25.9%	
Family history of prostatic disease					0.335
Yes	51	70.8%	21	29.2%	
No	241	76.3%	75	23.7%	
Have you ever gone to a doctor and/or urologist for prostate problems?					0.790
Yes	19	73.1%	7	26.9%	
No	273	75.4%	89	24.6%	
Has your doctor and/or urologist told you about the PSA test?					0.143^
Yes	5	55.6%	4	44.4%	
No	14	82.4%	3	17.6%	
Have you ever had a PSA test?					0.878
Yes	17	73.9%	6	26.1%	
No	275	75.3%	90	24.7%	
If it were you, would you have a PSA test?					0.363
Yes	164	73.5%	59	26.5%	
No	128	77.6%	37	22.4%	

## Discussion

The results of the current study revealed that Saudi Arabian participants demonstrated a significant lack of knowledge and awareness regarding PC and PSA testing. While most participants acknowledged that PC is the most common malignant tumor in men, nearly half stated they had learned about the condition, and a considerable percentage expressed a need for more information. These findings, which align with those of other studies conducted in the region, indicate a serious shortfall in public health education concerning PC. For example, Jarb AF et al. (2022) identified low awareness of PC and screening methods in a Saudi Arabian study, underscoring the necessity for targeted educational campaigns [9]. Further research in Saudi Arabia also indicated limited knowledge and awareness of PC and its diagnostic methods, as evidenced by studies conducted by Alothman AM et al. [6] and Ghunaim A et al. [10]. Additionally, studies in Egypt [11] and Jordan [12] found that most participants had poor knowledge and only fair attitudes toward PC examinations and screening practices. In contrast, a study in Jamaica showed that a vast majority of participants held a positive attitude toward PC screening and prevention while also demonstrating moderate knowledge of the disease [13]. Furthermore, a study in Turkey indicated that 88.4% of participants recognized that PC is treatable if diagnosed early [14]. Another investigation in Italy in 2017 found that 82.1% of participants reported having heard of PC, with 31.8% having learned about it from a doctor [8].

Global evidence on risk factors for PC supports the identification of risk factors such as smoking, a family history, and being over 50 years old [15]. The comparatively low level of knowledge regarding other risk factors, such as obesity and high-fat diets, suggests that Saudi Arabian public health messaging may need to be more thorough. This contrasts with research from Western nations, where widespread public health campaigns have raised awareness of lifestyle-related risk factors [16]. For instance, the vast majority of participants in a U.S. study recognized diet and obesity as significant risk factors for PC [17].

Given the importance of early detection in improving the prognosis of PC, it is concerning that so few participants were aware of PSA testing, and even fewer had been informed about it. This result aligns with other studies from the Middle East that found similarly low levels of awareness about PSA testing, including one conducted in the United Arab Emirates [18]. However, this sharply contrasts with findings from countries such as the United States, where widespread screening programs and physician recommendations have significantly increased awareness of PSA testing [19].

Relying on unofficial information sources, such as the internet and social media, rather than credible medical authorities like doctors, is a notable trend. This pattern is not limited to Saudi Arabia; similar behaviors have been observed in other developing countries, where social media is often a primary source of health information due to its accessibility [20]. The overall low level of knowledge among participants reflects findings from other studies in Saudi Arabia and the broader Middle East. For instance, a survey conducted by Almuhanna et al. (2018) showed that only a tiny percentage of Saudi men possessed adequate knowledge about PC, echoing the results of this study [21]. In contrast, research from high-income countries like Canada and Australia indicates higher levels of knowledge attributed to well-established public health campaigns and screening initiatives [22,23].

A majority of participants expressed support for PSA testing for asymptomatic men over 50, recognizing its importance in early detection and acknowledging that the risk of PC increases with age. Still, a sizable portion were uncertain about the benefits of PSA testing. Furthermore, fewer than one-third of participants expressed personal concern about developing PC, with many either disagreeing or feeling unsure about their risk. These results suggest that while there is a general understanding of age as a risk factor and the significance of early detection, targeted education remains necessary to address uncertainties and promote proactive screening, particularly among older men. Additional studies have shown positive attitudes and perceptions regarding PC and its screening methods, aligning with the current study's findings [24-26]. In terms of practice, the current study indicated a low commitment to PSA testing among participants, with the vast majority never having visited a doctor for prostate-related issues or undergone the test. Significant obstacles were identified as a lack of doctor recommendations, low perceived risk, fear of diagnosis, and doubts about the test's efficacy. A notable portion of participants remained apprehensive, even though most indicated they would be open to considering PSA testing for early detection.

Study limitations

This study has some limitations that should be acknowledged. First, the use of an online convenience sampling approach resulted in a sample predominantly composed of participants under the age of 30. This youthful age distribution limits the generalizability of the findings, particularly to older adult men, who are the primary risk group for PC and may have different levels of awareness. Although a subgroup analysis for men aged ≥50 years was suggested, the number of respondents in this age range was too small to perform a statistically meaningful analysis. Future research should employ targeted recruitment strategies or probability-based sampling to ensure adequate representation of older men. Second, the reliance on self-administered electronic questionnaires may introduce response bias, including the possibility that participants with higher education or health literacy were more likely to complete the survey. Additionally, as with any cross-sectional study, causality cannot be inferred. Despite these limitations, the study provides valuable preliminary insight into PC awareness within the sampled population and highlights areas where public health education may be strengthened.

## Conclusions

This study examined knowledge, attitudes, and practices regarding PSA testing and PC among Saudi Arabian participants, mainly from the Al Ahsa region. Findings revealed substantial gaps in awareness of PC risk factors, prevention, and the benefits of early detection. Key barriers included limited physician recommendations, low perceived risk, fear of diagnosis, and reliance on unofficial sources such as social media. Although many participants were willing to undergo PSA testing, overall knowledge was low, particularly among younger individuals. Given that most respondents were under 30 and a convenience sampling method was used, results should be interpreted cautiously and may not generalize to older men at higher risk with greater healthcare exposure. The study provides region-specific data, identifies education and employment as factors associated with knowledge, highlights social media as an important information source, and offers early insights into gaps that targeted education could address before men reach at-risk ages. These findings emphasize the need for culturally sensitive interventions, improved patient-provider communication, and broader public health campaigns. Future research should purposively recruit older participants and use mixed-methods approaches to better understand knowledge, attitudes, and practices across diverse populations.

## References

[1] Arafa MA, Farhat KH, Rabah DM (2015). Knowledge and attitude of the population toward cancer prostate Riyadh, Saudi Arabia. Urol Ann.

[2] Nakandi H, Kirabo M, Semugabo C, Kittengo A, Kitayimbwa P, Kalungi S, Maena J (2013). Knowledge, attitudes and practices of Ugandan men regarding prostate cancer. Afr J Urol.

[3] Makungu ML, Mweya CN (2023). Assessing knowledge, attitude and practice towards prostate cancer screening among males in Southwest Tanzania: a cross-sectional study. Cancer Treat Res Commun.

[4] Chisamba T, Maree JE, Jansen van Rensburg JJ (2023). Knowledge, attitudes and practices of Zimbabwean men relating to prostate cancer. Curationis.

[5] Musalli ZF, Alobaid MM, Aljahani AM, Alqahtani MA, Alshehri SS, Altulaihi BA (2021). Knowledge, attitude, and practice toward prostate cancer and its screening methods among primary care patients in King Abdulaziz Medical City, Riyadh, Saudi Arabia. Cureus.

[6] Alothman AM, Altamimi AF, Alhenaki AW, Almansour NM, Alhusaini AK, Alateeq F (2022). The knowledge and attitude towards prostate cancer and screening practices among males in Saudi Arabia. J Family Med Prim Care.

[7] Elyas A, Mahfouz MS, Suwaydi AZ (2024). Prostate cancer knowledge and attitude toward screening practices among men 40 and over in the Jazan region, Saudi Arabia. J Cancer Epidemiol.

[8] Morlando M, Pelullo CP, Di Giuseppe G (2017). Prostate cancer screening: Knowledge, attitudes and practices in a sample of men in Italy. A survey. PLoS One.

[9] Jarb AF, Aljuaid AK, Alghamdi SM, Almathami AA, Altawili AA, Alesawi A (2022). Awareness about prostate cancer and its screening in Medina, Jeddah, and Makkah, Saudi Arabia population. Urol Ann.

[10] Ghunaim AAA, Aljohani HS, Alharbi YA (2018). The extent of knowledge and awareness of prostate cancer screening among Saudi men aged more than 40 years. Egypt J Hosp Med.

[11] Mohamed NS, Asfari JM, Sambawa SM, Aljurf RM, Alsaygh KA, Arafah AM (2025). Awareness of prostate cancer and its screening tests in men in the Middle East: a systematic review and meta-analysis. J Family Community Med.

[12] Alqudah MA, Al-Samman R, Matalgah O, Abu Farhah R (2022). Early detection of prostate cancer: self-reported knowledge and attitude of physicians in Jordan. Inquiry.

[13] Morrison BF, Aiken WD, Mayhew R, Gordon Y, Odedina FT (2017). Prostate cancer knowledge, prevention, and screening behaviors in Jamaican men. J Cancer Educ.

[14] Turkan S, Doğan F, Ekmekçioğlu O, Çolak A, Kalkan M, Şahin Ç (2016). The level of knowledge and awareness about prostate cancer in the Turkish male and the relevant effective factors. Turk J Urol.

[15] Rawla P (2019). Epidemiology of prostate cancer. World J Oncol.

[16] Schröder FH, Hugosson J, Roobol MJ (2014). Screening and prostate cancer mortality: results of the European Randomised Study of Screening for Prostate Cancer (ERSPC) at 13 years of follow-up. Lancet.

[17] Smith RA, Andrews KS, Brooks D, Fedewa SA, Manassaram-Baptiste D, Saslow D, Wender RC (2019). Cancer screening in the United States, 2019: A review of current American Cancer Society guidelines and current issues in cancer screening. CA Cancer J Clin.

[18] Humaid Al-Shamsi S, Humaid Al-Shamsi A, Humaid Al-Shamsi M (2023). The perception and awareness of the public about cancer and cancer screening in the United Arab Emirates, a population-based survey. Clin Pract.

[19] Drazer MW, Huo D, Schonberg MA, Razmaria A, Eggener SE (2011). Population-based patterns and predictors of prostate-specific antigen screening among older men in the United States. J Clin Oncol.

[20] AlGhamdi KM, Moussa NA (2012). Internet use by the public to search for health-related information. Int J Med Inform.

[21] Almuhanna A, Alshammari S, Alsalman H (2018). Awareness of prostate cancer, screening and methods of managements in a hospital in Riyadh, Saudi Arabia. Egypt J Hosp Med.

[22] Loeb S, Carter HB, Berndt SI, Ricker W, Schaeffer EM (2011). Complications after prostate biopsy: data from SEER-Medicare. J Urol.

[23] Pinnock C, O'Brien B, Marshall VR (1998). Older men's concerns about their urological health: a qualitative study. Aust N Z J Public Health.

[24] Schulman C (2007). Assessing the attitudes to prostate cancer treatment among European male patients. BJU Int.

[25] Lepherd L, Graham C (2016). A positive attitude in prostate cancer challenges: finding hope and optimism. J Community Support Oncol.

[26] Berglund G, Nilsson S, Nordin K (2005). Intention to test for prostate cancer. Eur J Cancer.

